# Prevalence of posttraumatic stress disorder and associated factors among displaced people in Africa: a systematic review and meta-analysis

**DOI:** 10.3389/fpsyt.2024.1336665

**Published:** 2024-03-05

**Authors:** Fantahun Andualem, Mamaru Melkam, Girmaw Medfu Takelle, Girum Nakie, Techilo Tinsae, Setegn Fentahun, Gidey Rtbey, Tesfaye Derbie Begashaw, Jemal Seid, Lidiya Fasil Tegegn, Getachew Muluye Gedef, Desalegn Anmut Bitew, Tilahun Nega Godana

**Affiliations:** ^1^ Department of Psychiatry, College of Medicine and Health Science, University of Gondar, Gondar, Ethiopia; ^2^ Department of Psychiatry College of Medicine and Health Science, Wollo University, Dessie, Ethiopia; ^3^ Department of Nursing, College of Medicine and Health Science, Arsi University, Asella, Ethiopia; ^4^ Department of General Midwifery, College of Medicine and Health Science, University of Gondar, Gondar, Ethiopia; ^5^ Department of Reproductive Health, Institute of Public Health, College of Medicine and Health Science, University of Gondar, Gondar, Ethiopia; ^6^ Department of Internal Medicine, University of Gondar College of Medicine and Health Science, Comprehensive Specialized Hospital, Gondar, Ethiopia

**Keywords:** epidemiology, prevalence, posttraumatic stress disorder, PTSD, displaced people, refugees, internal displaced people, Africa

## Abstract

**Background:**

The number of people who have been displaced from their homes due to violence, conflict, and natural disasters. The displaced persons are vulnerable to PTSD; however, being women, individuals with lower socio-economic status and intense exposure to physical assault are more vulnerable. The reviews stated that the pooled prevalence of PTSD among refugees in high-income countries was higher than the general population. However, there has been no review done on PTSD among displaced persons in Africa. Therefore, the aim of this review was to summarise the most recent data evidence on the pooled prevalence of posttraumatic stress disorder and the pooled effect of associated factors on adult displaced people in Africa.

**Methods:**

We used an appropriate guideline for systematic reviews and meta-analyses reports, which is the Preferred Reporting Items for Systematic Reviews and Meta-Analyses (PRISMA). This review protocol was registered in PROSPERO (CRD42023411371). The publications were identified from PubMed/Medline, EMBASE, the Cochrane Library, Scopus databases, and other grey searches of Google Scholar and World Health Organisation (WHO) reports. The data was extracted in Microsoft Excel, and then it will be imported into STATA 11.0 for analysis.

**Results:**

We have included 10 studies conducted in African countries with 5287 study participants. In this meta-analysis, the pooled prevalence of PTSD among displaced people in Africa was 55.64 (95% CI: 42.76–68.41%). Further, in subgroup analysis regarding the study participants, the pooled prevalence of PTSD among internally displaced people and refugees was 56.35% and 54.04%, respectively. Among the associated factors, being female, unemployed, and depression were significantly related to PTSD among displaced people.

**Conclusions:**

In this review, the pooled prevalence of PTSD among displaced people in Africa was high. Demographic characteristics (female, single, and unemployed), substance use disorder, and depression were risk factors for PTSD among displaced people. This finding might help the stakeholders (mental health policy makers, administrators, and mental health professionals) to address the prevention, early screening, and management of PTSD among displaced people and to give attention to more vulnerable bodies.

**Systematic review registration:**

PROSPERO, identifier CRD42023411371.

## Introduction

The number of people who have been displaced from their homes due to violence, conflict, and natural disasters. In general, displaced people can be classified as migrants, refugees, or asylum seekers, as well as internally displaced persons (IDPs) (1). People who are compelled to leave their native country due to conflict, war, persecution, violations of human rights, and economic and political crises are known as refugees (2–4). Whereas, internally displaced persons (IDPs) are groups of people who have been compelled to leave their homes or residences due to an armed conflict, widespread violence, human rights abuses, or natural or man-made disasters but have stayed within the borders of their own country (5, 6).

The United Nations Human Rights Commission (UNHRC) estimates that almost 80 million people were ejected from their homes forcibly (7). They were divided into three groups: 45.7 million internally displaced people, 29.6 million externally displaced people, and 4.2 million people seeking asylum. The number of people who have been forcibly displaced has increased by 75% globally during the past two decades (8). The number of refugees has significantly increased in recent years (9), with 22.55 million refugees reported worldwide (10). According to GRID, there were around 40 million internally displaced individuals as a result of conflict (11), while additional sources indicated that there were 55 million internally displaced individuals globally, of which more than 87.2% were uprooted due to conflict and violence and 12.8% were uprooted owing to natural catastrophes (12).

The majority of displaced individuals are found in developing nations (13, 14). There are 12.6 million displaced people in Africa as a result of conflict, and sub-Saharan Africa is home to more than 26% of the world’s refugee population (13). 11.8 million people were internally displaced due to conflict and violence in Sub-Saharan Africa, according to the Global Report on Internal Displacement (GRID) (15). Ethiopia had 3.2 million IDPs in 2019, which is the second-highest figure in Africa behind the Democratic Republic of the Congo, which had more than 5 million IDPs in 2019 (16). According to a UNHCR report, 42% of all IDPs worldwide lived in Africa. Nearly 12.6 million individuals were internally displaced in Africa as a result of conflict in 2016 (17).

Displaced persons are more susceptible to traumatic incidents such as physical or sexual assault, torture, threats of death, murder, the death of loved ones, and economic losses (18–21). Generally inadequate recompense for basic human needs and the breakdown of the public order (22) may alter their biopsychosocial domains (23, 24), and they can affect the biopsychosocial domains (23), which can result in post-traumatic stress disorder (25–27).

Post-Traumatic Stress Disorder (PTSD) is holding attention as a mental disorder after observations of battle fatigue, shell shock, and soldiers’ hearts in both World Wars I and II (28). PTSD is marked by increased stress and anxiety following exposure to a traumatic or stressful event, which may be a man-made or natural disaster. Exposure to a traumatic event might mean directly experiencing, witnessing, and learning that the traumatic event(s) occurred to a close family member. As a result, traumatised individuals manifest intrusive distress (memories or dreams) and flashbacks of the traumatic event(s), and then they try to avoid being reminded, persistent avoidance of stimuli, and negative alterations in cognitions and mood associated with the traumatic event(s) (28–34).

As the World Health Organisation’s (WHO) mental health surveys report, the lifetime prevalence of PTSD ranged from 3.9% to 5.6% (35), and in the United States of America (USA), it was 7% to 8% (34). The prevalence of trauma exposure and PTSD in low-income countries was 76% and 11.2%, respectively (36). The displaced persons are vulnerable to PTSD; however, being women, individuals with lower socio-economic status and intense exposure to assault are more vulnerable (28).

The reviews stated that the pooled prevalence of PTSD among refugees in high-income countries or Western countries was higher than the general population (37–39), 29% and 37% for diagnosed and self-reported PTSD, respectively (40), and another review reported that 8% to 37.2% (41). However, there has been no review done on PTSD among displaced persons or separately among refugees and internally displaced persons in Africa. Indeed, the PTSD status of displaced persons has been the topic of a large number of studies, with a large variation in reported prevalence rates from 18.4% (42) to 78% (15).

A systematic review in high-income counties indicates a challenging and persisting disease burden in refugees due to anxiety, mood disorders, and PTSD (40). PTSD in refugees is leading to adverse effects on their children’s mental health due to harsh parenting styles (43). In addition, PTSD, if left untreated, may often complicate other adverse mental health outcomes, presenting as comorbid depression, anxiety, and substance abuse disorders (28, 34, 44). Other reviews revealed that there are limited practices regarding assessment (45) and treating trauma-exposed people (46).

Identifying risk factors and the pooled prevalence of PTSD among displaced persons could help health care professionals and policymakers plan health promotion, prevention, and early intervention programmes (47, 48). However, per our search, there has been no systematic review or meta-analysis of the epidemiology of PTSD among displaced people in Africa. The aim of this systematic review and meta-analysis is to summarise the most recent data on adult displaced persons in Africa from January 2000 to April 2023, covering the first quarter of the twenty-first century, in order to fill up this research vacuum by providing answers to the following questions:

1. What is the pooled prevalence of PTSD among displaced people?
2. What are the factors associated with PTSD among displaced people?

## Methods and materials

### Design

The publications were searched by PubMed/Medline, EMBASE, Cochrane Library, Scopus, HINARI, PsycINFO, African Journals Online (AJOL), and other grey publications were searched by Google Scholar and World Health Organisation (WHO) reports. The following search items (Mesh) were used (“prevalence” OR “epidemiology” AND “post-traumatic stress disorder” OR “PTSD” AND “associated factors” OR “risk factors” OR “determinants” AND “displaced people” OR “refugees” OR “internal displaced people” AND “Africa”) (**Supplementary File 1**). The data was extracted from articles. Research reports were included from January 2000 to April 2023, and the studies were evaluated for their eligibility, and then they were included in the meta-analysis using prepared eligibility assessment criteria. We used an appropriate guideline for systematic reviews and meta-analyses reports, which is the Preferred Reporting Items for Systematic Reviews and Meta-Analyses (PRISMA) (49) (**Supplementary File 2**). This review protocol was registered in PROSPERO (CRD42023411371).

### Eligibility criteria

The studies meeting the following criteria were part of this systematic review and meta-analysis: the studies conducted in African countries and all relevant observational studies (cross-sectional, cohort, and case-control studies). And also, adults (>=18 years old), published in English or having an English translation, published and unpublished articles were considered. Searching was performed from February 2023 to April 2023, and articles available online from January 2000 to April 2023 were considered. But studies that could not be fully accessed (conference abstracts) after a request was made from their author by email were excluded from the study because we were unable to assess the quality of each article in the absence of full reports.

**Figure 1:**
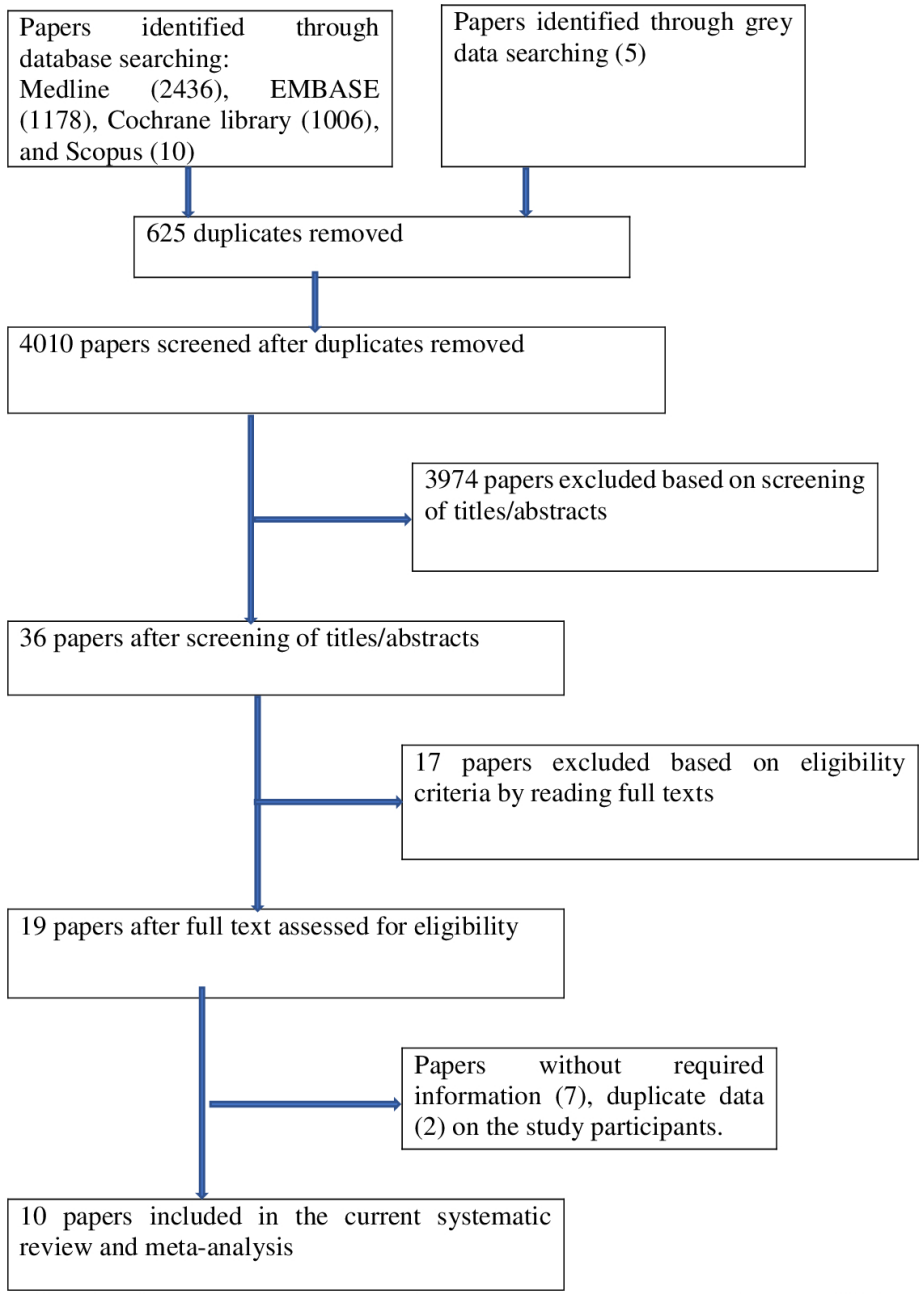
PRISMA flowchart of review search on the prevalence and associated factors of PTSD among displaced people.

Based on inclusion criteria, we first screened titles and abstracts of articles retrieved from the initial search, and then, following the selection of pertinent research, the entire text was examined. As seen in **Figure 1**, we did not include any articles with no interesting variables in our study.

### Data extraction

MM and GM independently extracted all the necessary data from the articles using a standardised data extraction format. The data extraction format included the following items: the first author’s name, publication year, country where the study was conducted, a screening tool used to examine PTSD, number of participants, prevalence of PTSD, and associated factors with PTSD. The data extraction format was in the form of a two-by-two table. A cross-check was done by MM and GM following searches. If contrasting results occurred between the two authors during data extraction, they were discussing ways to achieve consensus and double extraction with other authors. We have done a sensitivity analysis to assess the robustness of the meta-analytic results.

### Tools and validity

Among the ten papers reporting estimates of the prevalence of PTSD were:

1. Three were assessed using the Harvard trauma questionnaire (HTQ) (49) designed by the Harvard Programme in Refugee Trauma at Massachusetts General Hospital for the diagnosis of symptomatic PTSD. The PTSD section consists of 16 questions based on the diagnostic criteria of the Diagnostic and Statistical Manual for Mental Disorders, Fourth Edition (DSM-IV) (50), but all of them were measured at different cut-off points.
2. Two papers were assessed using the Post-Traumatic Stress Disorder Checklist for DSM-5 (PCL-5). The PCL-5 is a standardised instrument and a self-report rating scale for assessing the 20 DSM-5 symptoms of PTSD (51). A total score was computed by adding the 20 items, so that possible scores range from 0 to 80 with a 5-point Likert scale (0–4) with a cut-off point of >=33 (51). The validity and reliability of the PCL-5 have been tested and proven on displaced people and refugees in a number of countries, for example, Iraq (Cronbach’s alpha =0.85) (52) and Zimbabwe (Cronbach’s alpha =0.92) (53).
3. The International Neuropsychiatric Interview 7 (MINI-7). The MINI is a short, structured diagnostic interview compatible with the Diagnostic and Statistical Manual of Mental Disorders 5. It was designed for clinical practice, research in psychiatric primary care settings, and epidemiological surveys (54, 55). The MINI was chosen based on the validity and reliability demonstrated in different populations in Brazil and Japan (56, 57).
4. The Impact of Event Scale-6 (IES-6) measures the level of exposure to traumatic events (58). The questions were measured on a four-point severity scale of 0–4, with a total maximum score of 24. Scores for each respondent were summed up, and individuals with a total score of 10 were considered symptomatic for PTSD.
5. The Impact of Event Scale-Revised (IES-R) (59) is a 22-item self-report measure that assesses subjective distress arising from traumatic events and experiences. The 22 items are rated on a 5-point scale (0 -4). A cut-off score of >=33 was used to identify PTSD (60).
6. The primary care PTSD screen (PC-PTSD) is a four-item screening instrument that was designed for use in primary care and other settings and is currently used to screen for PTSD. It includes an introductory sentence to alert respondents to traumatic events. The results of the PC-PTSD should be considered “positive” if a patient answers “yes” to any three items (61).
7. The National Stressful Events Survey PTSD Short Scale (NSESSS) was used.

### Quality appraisal

The quality of the research reports included in this review was assessed using the Joanna Briggs Institute (JBI) for cross-sectional study quality assessment (62). For this quality assessment tool, it included the following components: the methodological quality of the study, comparability of included studies, and quality of original articles with respect to statistical analysis. All authors independently evaluated the quality of the original research using JBI. The tool has a total of 9 scores, and articles with medium and high quality (articles that score 5 and above out of a 9-point scale) were included in this review for analysis. If there were any discrepancies between authors during the quality assessment of the included studies, they were solved by taking the average of each author.

### Data processing and analysis

The data was extracted in Microsoft Excel, and then it was imported into STATA 11.0 for analysis. The original studies were shown by using texts, tables, and forest plots. The standard error of prevalence for each primary or original study was analysed using the binomial distribution. The prevalence of the original studies was checked for heterogeneity using a heterogeneity I^2^ test (63). We used a random-effects meta-analysis model to estimate Der Simonian and Laird’s pooled effect of PTSD. And we made a leave-one-out sensitivity analysis to identify the possible source of heterogeneity in the pooled meta-analysis of the prevalence of PTSD among displaced people in African countries. Publication bias was detected using funnel plot analysis and Egger-weighted regression tests. A p value of less than 5% significance in the Egger test was considered to have statistically significant publication bias (64, 65). Subgroup analyses were carried out to determine if there was any heterogeneity in the results. Predictors of heterogeneity were: study country, year of publication, study participants (whether internal displaced people or refugees), assessment tool of PTSD, type of study design, number of study participants, prevalence of PTSD among participants, females’ prevalence of PTSD among the participants, and methodological quality.

## Results

### Study identification

There were 4630 publications identified in database searches, and another 5 records were added through grey searches. Among them, 625 papers were removed due to duplicates; 3974 papers were excluded based on screening of titles or abstracts; 17 papers were excluded based on eligibility criteria by reading full texts; and 9 papers were excluded due to (without required information) 7 and duplicate data 2. Finally, a total of 10 studies were involved in this systematic review and meta-analysis (**Figure 1**).

### Characteristics of included studies

A total of 10 studies conducted in African countries, including 5287 study participants, were included in the current review. Among the 10 studies included, three were from Ethiopia (12, 42, 66), two from Nigeria (15, 67) and Uganda (68, 69), and one from Kenya (70), Somalia (71), and Zambia (72). Regarding the study design of the included studies, ten of them were cross-sectional study designs. PTSD was assessed by HQT in three studies, PCL-5 in two studies, and each of NSESSS, MINI-7, IES-6, PC-PTSD, and IES-R in one study (**Table 1**).

**Table 1:** Characteristics of included studies on PTSD among displaced people in Africa.

First author name, Year	Country	Participants	Study design	Tool	SZ	P, %	F, %	QS	Associated factors: [AOR (95% CI)]
Achille MB, 2020 (69)	Uganda	Refugees	Cross-sectional	MINI-7	387	67	66	9	SUDs:[5.13(2.32, 11.33)]; MDD:[4.04(2.24, 7.30)], GAD: [3.27(1.85, 5.76)]
Bayard R, 2008 (68)	Uganda	IDPs	Cross-sectional	HTQ	1210	54	66.2	9	Female:[2.2(1.67, 2.9)]; Single:[2.06(1.49, 2.84)]
Belay M, 2023 (12)	Ethiopia	IDPs	Cross-sectional	PCL-5	406	67.5	52.6	9	Unemployed:[2.09(1.24, 3.54)]
Deborah OA, 2020 (15)	Nigeria	IDPs	Cross-sectional	IES-6	1200	78	NR	8	…
Derebe M, 2020 (66)	Ethiopia	IDPs	Cross-sectional	PCL-5	625	58.4	61.1	9	Female:[2.40(1.6, 3.4)]; Depression:[2.60(1.20, 3.90)]
Josephine NM, 2018 (70)	Kenya	IDPs	Cross-sectional	NSESSS	139	62.1	62.8	7	…
Mustafa A, 2023 (71)	Somalia	IDPs	cross-sectional	HTQ	399	32	NR	9	Unemployed:[1.79(1.06, 3.04)]
Taiwo LS, 2014 (67)	Nigeria	IDPs	Cross-sectional	HTQ	258	42.2	NR	8	Depression:[3.53(1.66, 7.48)]
Teferi GG, 2022 (42)	Ethiopia	Refugees	Cross-sectional	PC-PTSD	396	18.4	NR	8	…
Victor M, 2021 (72)	Zambia	Refugees	Cross-sectional	IES-R	267	76.8	50.7	8	…

SZ, sample size; P, participants’ prevalence of PTSD; F, females’ prevalence of PTSD, NR, not reported; QS, quality score; AOR, adjusted odd ratio; CI, confidence interval.

### Study quality appraisals

The Joanna Briggs Institute (JBI) was used to assess the quality of the studies. All the studies involved in this review have good quality (JBI score >6) (**Supplementary File 3**).

### Meta- analysis

**Figure 2:**
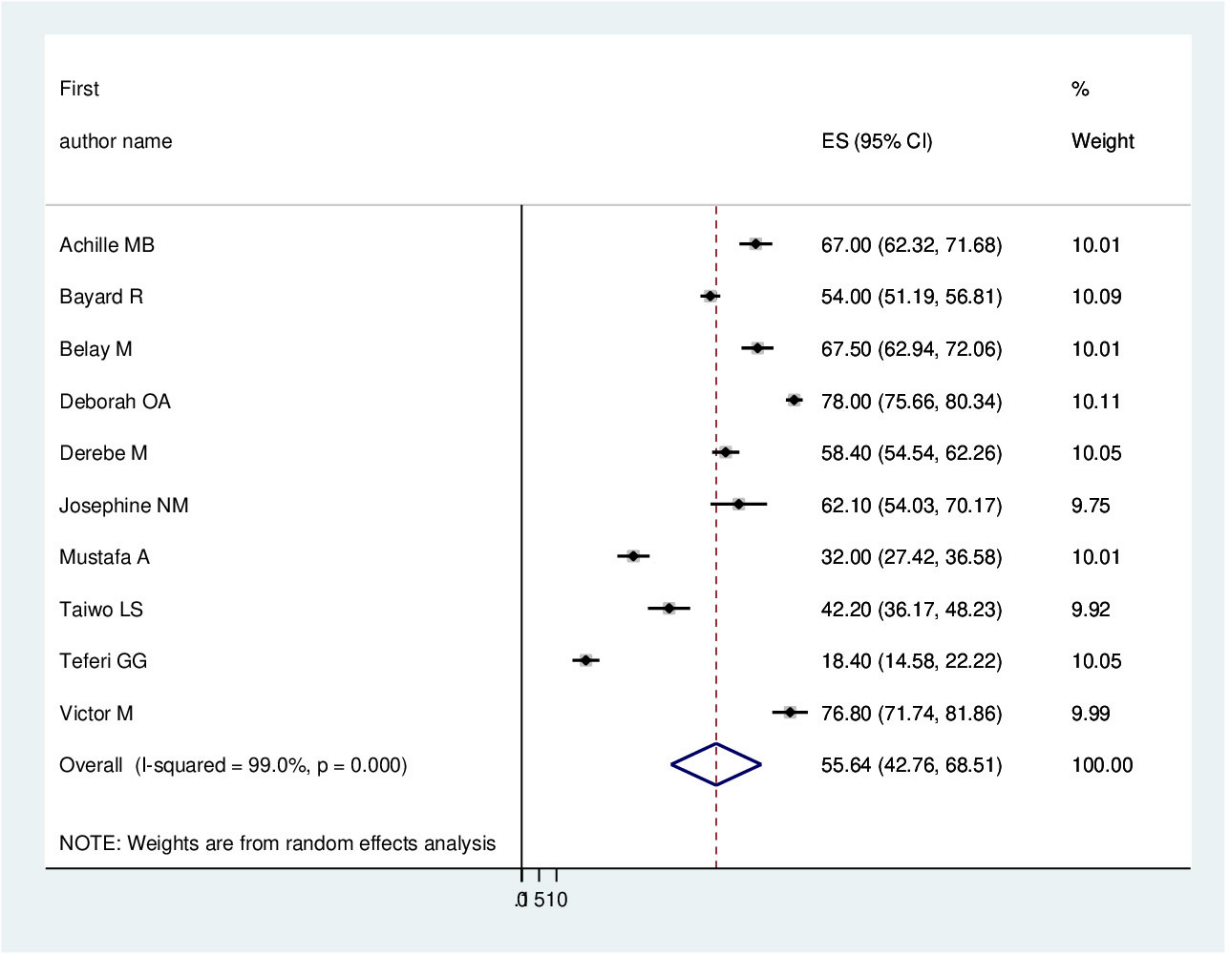
Forest plot of the pooled prevalence of PTSD among displaced people in Africa.

The pooled prevalence of PTSD among displaced people in Africa was 55.64 (95% CI: 42.76–68.41%) (**Figure 2**). Due to apparent heterogeneity across the studies, we have used a random effect model while conducting a meta-analysis (I^2^ = 99%, p < 0.001).

### Publication bias

**Figure 3:**
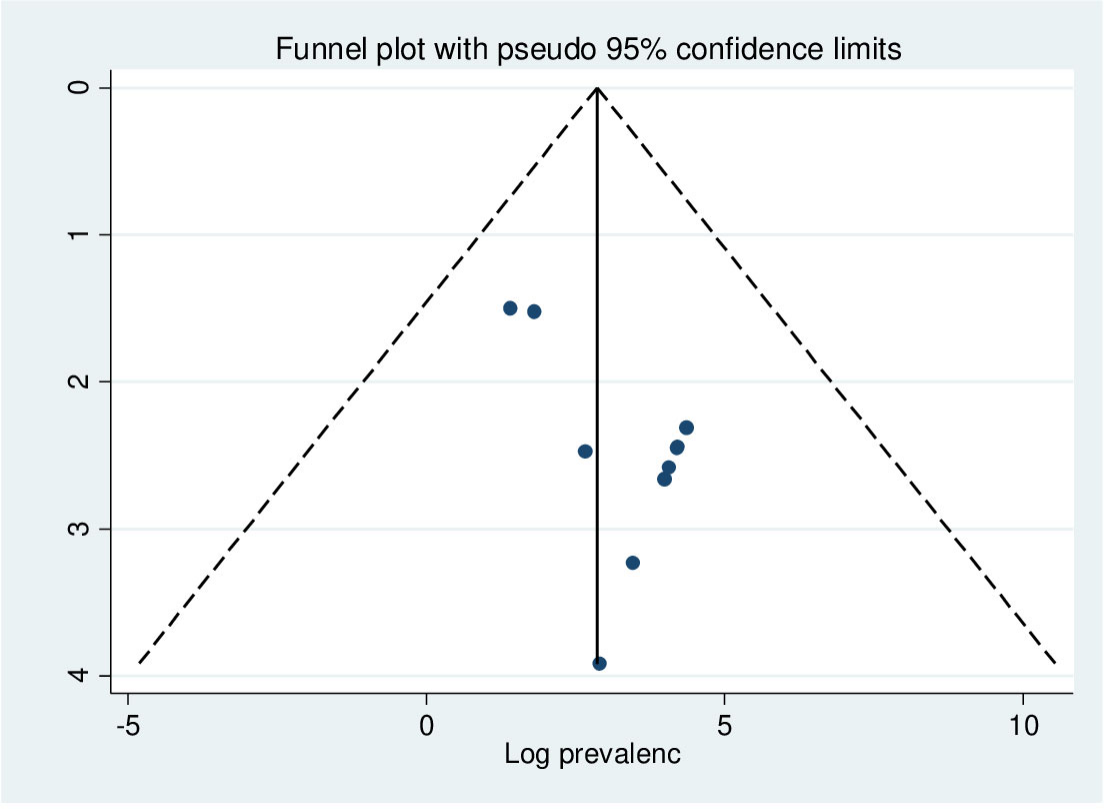
Funnel plot of PTSD among displaced people in Africa.

In this study, as shown in **Figure 3**, a funnel plot was symmetrical, which indicates the absence of publication bias, and Egger’s regression test (P = 0.403) strengthened it (**Table 2**).

**Table 2:** Egger’s test of PTSD among displaced people in Africa.

Std_-_Eff	Coef.	Std. Err.	T	P>t	95% Conf Interval	
Slope	75.11417	19.71753	3.81	0.005	29.64546	120.5829
Bias	-8.827404	9.987236	-0.88	0.403	-31.85801	14.2032

### Subgroup analysis

**Figure 4:**
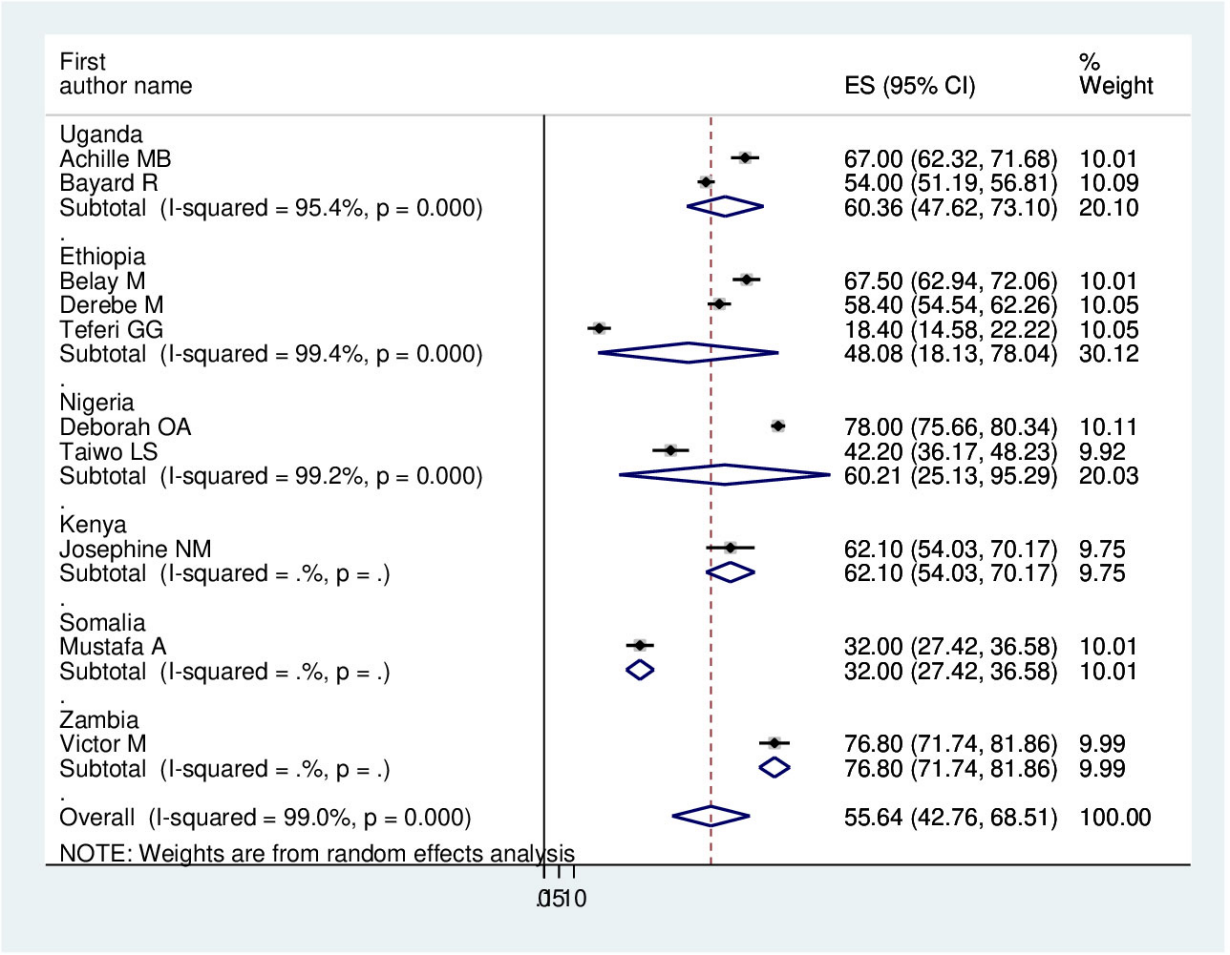
Forest plot on subgroup analysis by study country of the pooled prevalence of PTSD among displaced people in Africa.

**Figure 5:**
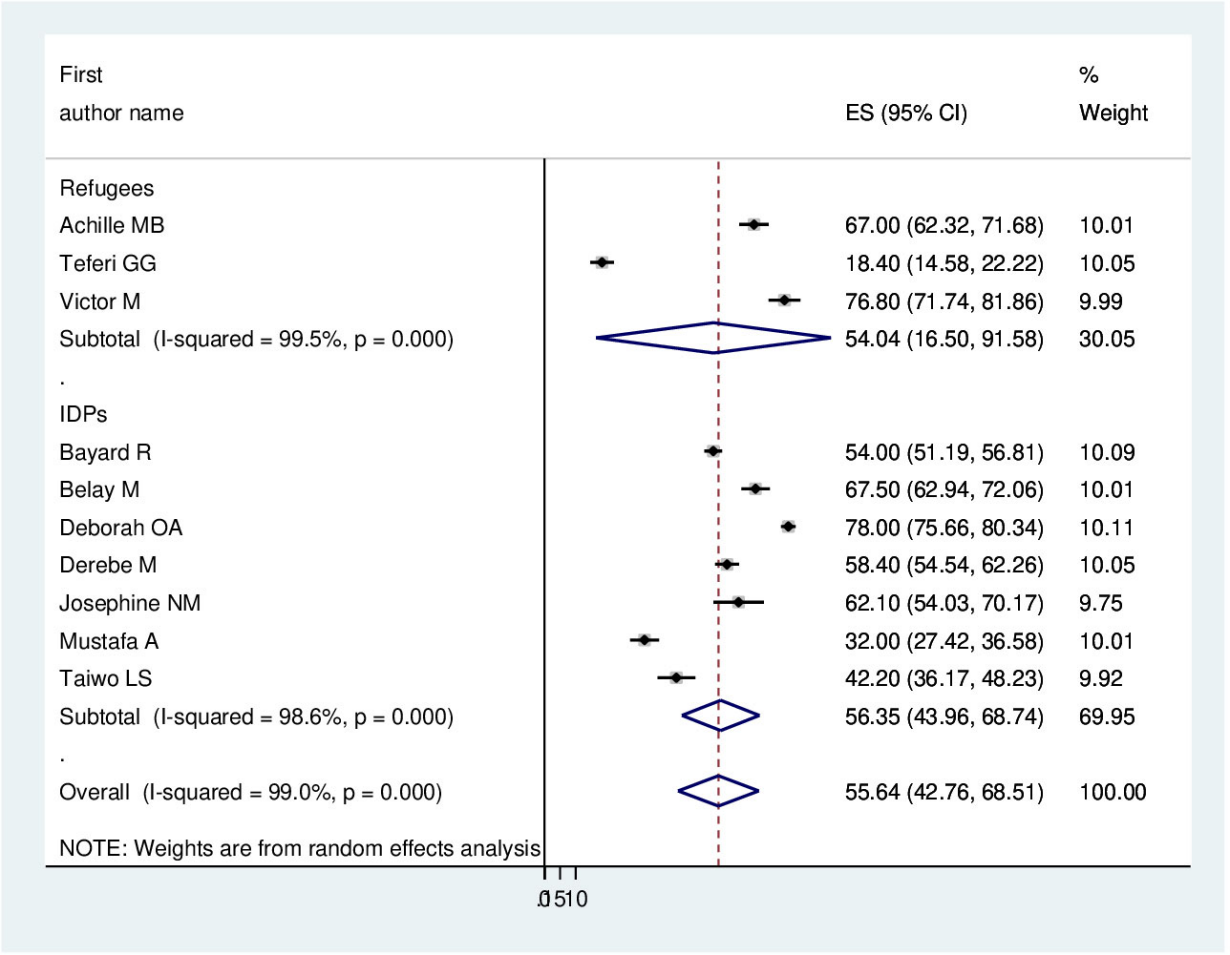
Forest plot on subgroup analysis by type of participants of the pooled prevalence of PTSD among displaced people in Africa.

**Figure 6:**
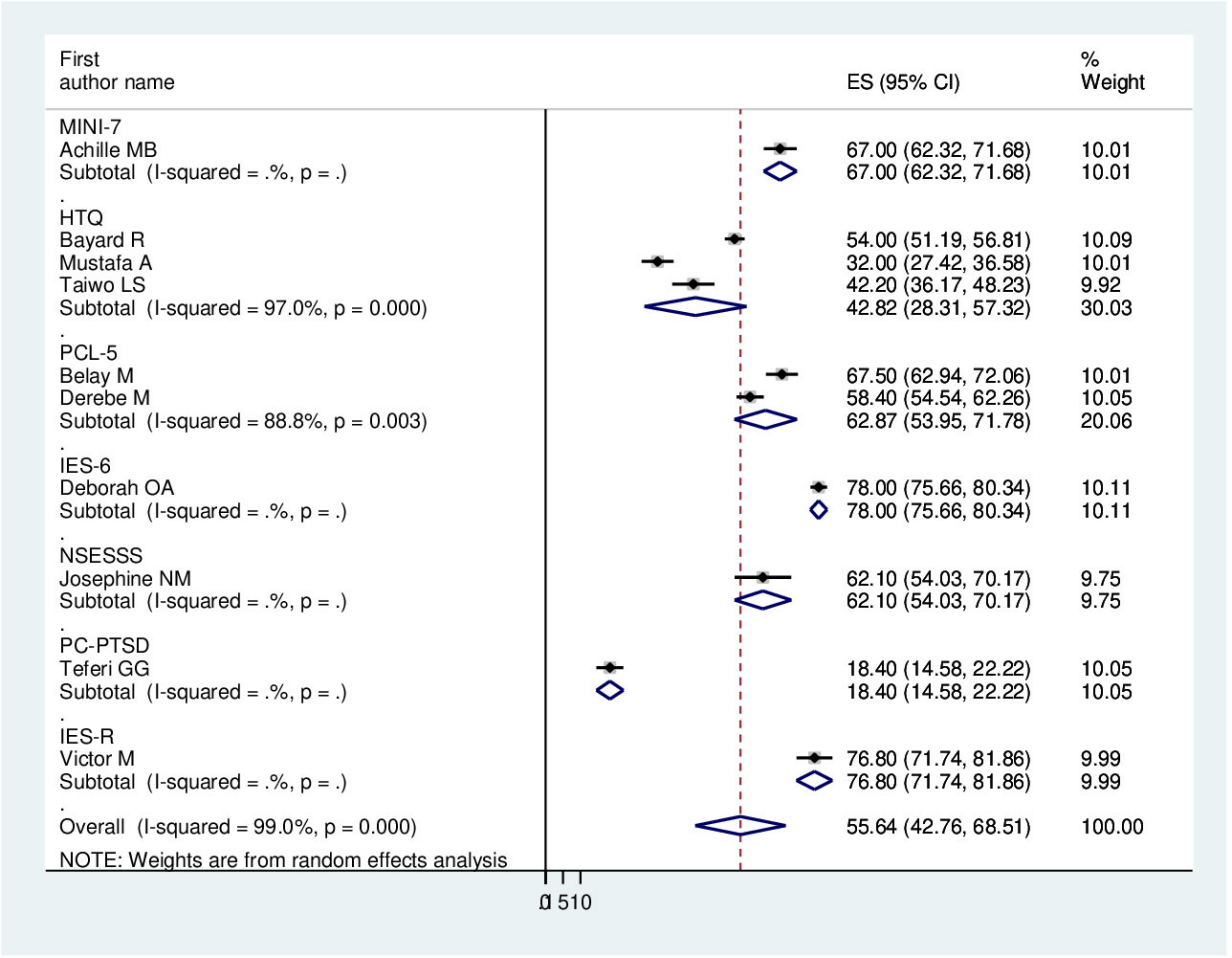
Forest plot on subgroup analysis by assessment tool of the pooled prevalence of PTSD among displaced people in Africa.

As previously discussed, the presence of heterogeneity was confirmed, and as a result, subgroup analysis was conducted based on the study country, type of participants, and assessment tool. A higher pooled prevalence of PTSD was found among studies conducted in Uganda (60.36%, I^2^ = 95.4%, p ≤0.001), Nigeria (60.21%, I^2^ = 99.2%, p ≤0.001), and Ethiopia (48.08%, I^2^ = 99.4%, p ≤0.001). Regarding the study participants, the pooled prevalence of PTSD among IDPs and refugees was 56.35% (I^2^ = 98.6%, p ≤0.001) and 54.04% (I^2^ = 99.5%, p ≤0.001), respectively. In addition, the pooled prevalence of PTSD on the assessment tool was 42.82% (I^2^ = 97%, p ≤0.001) for HTQ and 62.87% (I^2^ = 88.87%, p ≤0.001) for PCL-5. Therefore, this result showed there is high heterogeneity among subgroup analyses, as indicated by I^2^ (88.8 and above), which indicates the need to conduct the sensitivity test (P ≤0.003) (**Figures 4**–**6**).

### A leave-out-one sensitivity analysis

The sensitivity analysis was done to check the heterogeneity of those studies by omitting one author or one study step by step to check the effect of each study on the overall prevalence of PTSD in this review. As evidenced by the results, all the values are within the estimated 95% CI, which indicates the omission of a single study had no significant difference in the prevalence of this systematic review and meta-analysis (**Table 3**).

**Table 3:** Sensitivity analysis of PTSD among displaced people in Africa.

Study omitted	Estimate 95% CI	Heterogeneity	
		I^2^ (%)	P- value
Achille MB	54.37 (40.26-68.48)	99.1	0.000
Bayard R	55.82 (40.63-71.01)	99.1	0.000
Belay M	54.32 (40.19-68.44)	99.1	0.000
Deborah OA	53.11 (40.68-65.55)	98.6	0.000
Derebe M	55.33 (40.82-69.83)	99.1	0.000
Josephine NM	54.94 (41.17-68.70)	99.1	0.000
Mustafa A	58.27 (45.13-71.40)	99	0.000
Taiwo LS	57.12 (43.39-70.84	99.1	0.000
Teferi GG	59.81 (49.61-70.01)	98.3	0.000
Victor M	53.29 (39.57-67.00)	99.1	0.000

*CI, confidence interval.

### Associated factors analysis

**Figure 7:**
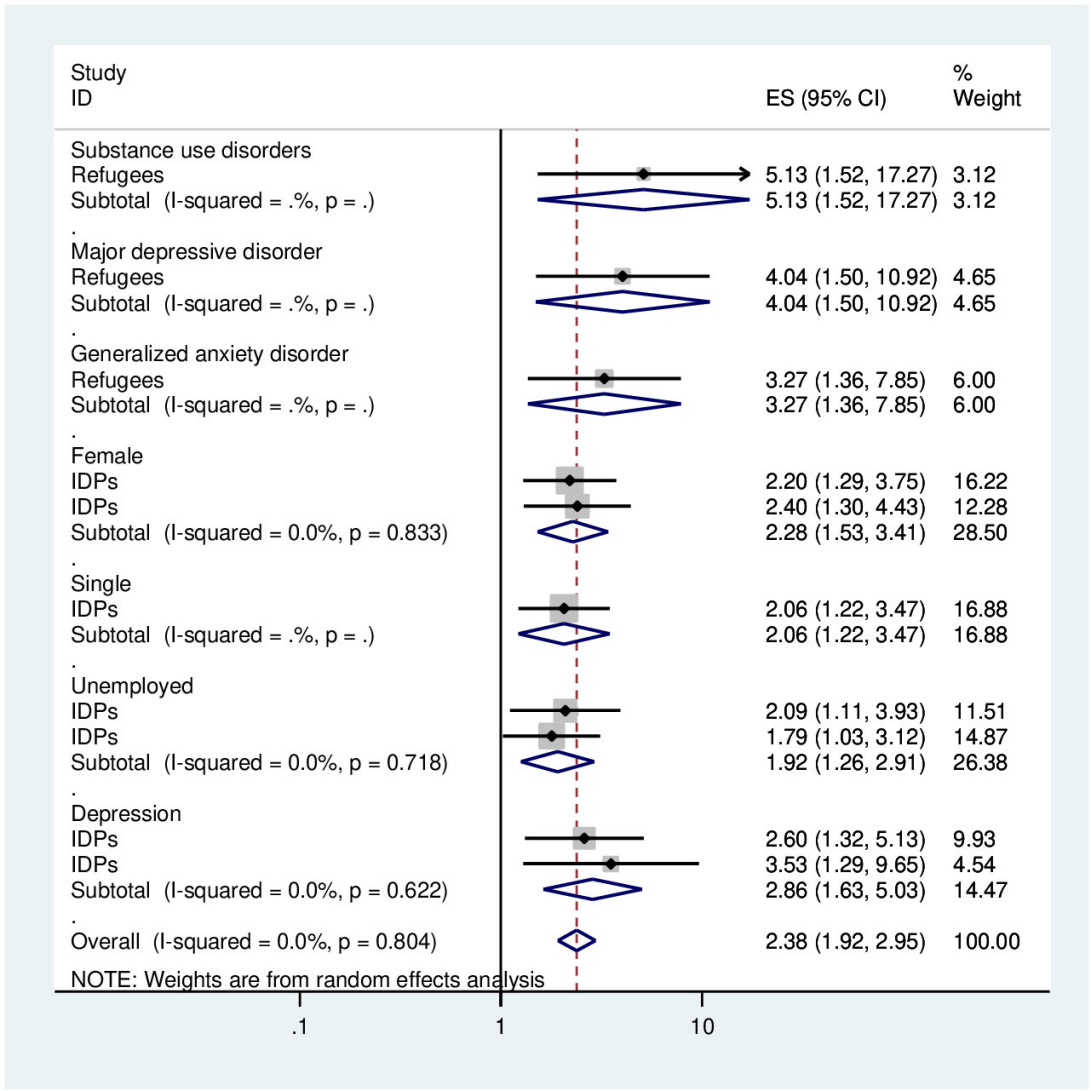
Forest plot showing different associated factors of PTSD among displaced people in Africa.

**Table 1** presents the significant variables linked to post-traumatic stress disorder (PTSD) in adult African-displaced individuals, based on individual studies assessed using logistic regression and an adjusted odd ratio. Significant variables related to PTSD in Africa have been described in this section. In terms of demographic characteristics, being female (AOR = 2.28, 95% CI: 1.53–3.41) and being unemployed (AOR = 1.92, 95% CI: 1.26-2.91) with two previous studies and within this review were significantly associated with PTSD. Regarding clinical factors, depression (AOR = 3.18, 95% CI: 2.05–4.95) with two previous studies and within the current study was significantly linked with PTSD (**Figure 7**). These results were obtained from the pooled analysis for these factors and in cases where two or more publications were present.

## Discussions

This systematic review and meta-analysis synthesised 10 studies investigating the prevalence and associated factors of PTSD among 5287 displaced people in Africa, of whom 2942 had been screened for PTSD. In this review, the pooled prevalence of PTSD among displaced people in Africa was 55.64 (95% CI: 42.76–68.41%). This result was in line with the study done among refugees in Germany (73) and among resettled Afghan refugees in Australia (47). However, it was higher than the study conducted in Ethiopia (74), in African countries (75), in Western countries (38, 40, 41), in the World Mental Health Survey (76), and in the Global Burden of PTSD (77). This gap might be related to the differences in study participants’ residency, type of participants, sample size, study design, and assessment tool.

This review covered six countries, but 13 studies in Ethiopia (74) with 5874 adult research participants were included. Eleven publications using a sample size of 7078 survivors of traffic accidents were included in a systematic review and meta-analysis conducted in Africa (75). Cross-sectional, longitudinal, and case-control research designs were included. On the other hand, this study used cross-sectional study methods and included studies on refugees and internally displaced people. According to the review, there were 25 surveys conducted with 3936 respondents about refugees and asylum seekers in Germany (38) and 66 publications with 14,882 respondents about refugees in high-income countries (40). However, this review’s participants were IDPs and refugees living in African nations. Another review revealed that six studies with a sample size of 1,912 participants were conducted among Iraqi refugees living in western countries (41). The instruments utilised to assess the participants’ PTSD—PCL-M, PC-PTSD, CAPS, CIDI, HTQ, and DSM-IV—were employed. The World Mental Health Survey indicates that 18 surveys, including 661 respondents, were conducted for disaster-related PTSD. The instruments employed in these surveys were CIDI, DSM-IV, and the Structured Clinical Interview for DSM-IV (SCID) (76). Using the PSS-I, CIDI, MINI, SIQ, and CAPS tools, 22 surveys with 15,420 adult participants in war zones indicated the worldwide burden of PTSD (77). In contrast, the MINI-7, HTQ, PCL-5, IES-6, NSESSS, and PC-PTSD were the instruments utilised in this review. As expressed under the validity of tool assessment, NSESSS has no more explanation about the number of items, cut-off points, or its sensitivity and specificity. So, it could result in differences in screening for PTSD regarding the assessment tool. In other aspects, this result was greater and in line with the findings from Earth Quick Survivors (range: 4.10–67.07%) that were reported (78). The meta-analysis was conducted to determine adult survivors’ PTSD connected to earthquakes. 56,722 people participated in the study, which comprised 37 papers, and different instruments were employed.

In this study, regarding subgroup analysis, the pooled estimate of PTSD among displaced people was higher in Uganda (60.36%, 95% CI: 47.62–73.1%) and Nigeria (60.21%, 95% CI: 25.13–95.29%) compared with Ethiopia (48.08%, 95% CI: 18.13–78.08%). The possible explanation could be the difference between the assessment tool and the sample size. Further, we have used the type of study participants; the pooled prevalence of PTSD among IDPs and refugees was very similar (56.35%, 95% CI: 43.96–68.74%) and (54.04%, 95% CI: 16.5–91.58%), respectively.

Our review identified that being female and unemployed were significantly associated with PTSD among displaced people. Among the psychosocial factors of PTSD are being female and unemployed (28–30). This result was that being female (79, 80)and unemployed (79) were significantly associated with PTSD, as supported by the review studies. In addition to demographic characteristics, depression is a risk factors and/or comorbid with PTSD (28–30). Therefore, our finding confirmed that depression was significantly associated with PTSD among displaced people.

## Strengths and limitations

This work could foster new views on how to manage the healthcare system or mitigate the effects of PTSD on quality of life, providing more information about how common PTSD is among those who have been uprooted. Additionally, the evaluation of many moderators was regarded as one of our study’s good points. Whereas, the limitation of this review is that it included only studies published in English that were cross-sectional studies since there were no studies conducted with other study designs and a small number of articles were included. In addition, most studies used very different instruments to screen for PTSD; therefore, it is difficult to compare and summarise the results.

## Conclusion

In this review, the pooled prevalence of PTSD among displaced people in Africa was high. Demographic characteristics (female, single, and unemployed), substance use disorder, and depression were risk factors for PTSD among displaced people. This finding might help the stakeholders (mental health policy makers, administrators, and mental health professionals) to address the prevention, early screening, and management of PTSD among displaced people and to give attention to more vulnerable bodies. Future research that focuses on a more accurate diagnosis should include more representative samples, or rather, a cross-sectional study design.

## Data availability statement

The original contributions presented in the study are included in the article/**Supplementary Material**. Further inquiries can be directed to the corresponding author.

## Author contributions

FA: Conceptualization, Data curation, Methodology, Software, Writing – original draft, Writing – review & editing. MM: Data curation, Writing – review & editing. GT: Writing – review & editing. GN: Data curation, Writing – review & editing. TT: Writing – review & editing. SF: Writing – review & editing. GR: Writing – review & editing. TD: Writing – review & editing. JS: Writing – review & editing. LF: Writing – review & editing. GM: Writing – review & editing. DA: Writing – review & editing. TG: Writing – review & editing.
