# Supplementary material for: Prevalence of posttraumatic stress disorder and associated factors among displaced people in Africa: a systematic review and meta-analysis
Source: Front Psychiatry. 2024 Mar 5;15:1336665. doi: 10.3389/fpsyt.2024.1336665 (PMC10956696; doi:10.3389/fpsyt.2024.1336665)
Supplement: Supplementary file 1 [file DataSheet_1.pdf]

Search strategy for databases

Research strategy in Medline:

(((('prevalence'/exp OR 'prevalence' OR 'epidemiology'/exp OR 'epidemiology') AND ('post-traumatic stress disorder'/exp OR 'post-traumatic stress disorder') OR 'ptsd'/exp OR 'ptsd') AND 'associated factors' OR 'risk factors'/exp OR 'risk factors' OR 'determinants'/exp OR 'determinants') AND 'displaced people' OR 'refugees'/exp OR 'refugees' OR 'internal displaced people') AND ('africa'/exp OR 'africa') AND [medline]/lim

Research strategy in EMBASE:

(((('prevalence'/exp OR 'prevalence' OR 'epidemiology'/exp OR 'epidemiology') AND ('post-traumatic stress disorder'/exp OR 'post-traumatic stress disorder') OR 'ptsd'/exp OR 'ptsd') AND 'associated factors' OR 'risk factors'/exp OR 'risk factors' OR 'determinants'/exp OR 'determinants') AND 'displaced people' OR 'refugees'/exp OR 'refugees' OR 'internal displaced people') AND ('africa'/exp OR 'africa') AND [medline]/lim AND [embase]/lim

Research strategy in Cochrane library:

(("prevalence" OR "epidemiology" AND "post-traumatic stress disorder" OR "PTSD" AND "associated factors" OR "risk factors" OR "determinants" AND "displaced people" OR "refugees" OR "internal displaced people" AND "Africa")) in Title Abstract Keyword

Research strategy in Scopus:

TITLE-ABS-KEY (("prevalence" OR "epidemiology" AND "post-traumatic stress disorder" OR "PTSD" AND "associated factors" OR "risk factors" OR "determinants" AND "displaced people" OR "refugees" OR "internal displaced people" AND "Africa"))
