# Supplementary material for: Prevalence of posttraumatic stress disorder and associated factors among displaced people in Africa: a systematic review and meta-analysis
Source: Front Psychiatry. 2024 Mar 5;15:1336665. doi: 10.3389/fpsyt.2024.1336665 (PMC10956696; doi:10.3389/fpsyt.2024.1336665)
Supplement: Supplementary file 3 [file DataSheet_3.docx]

**Table S 1 (Supplementary File 3):** Quality assessment of Post-Traumatic Stress disorder and its associated factors among displaced people in Africa included studies in this systematic review and meta-analysis.

| First author name (year) | Q1 | Q2 | Q3 | Q4 | Q5 | Q6 | Q7 | Q8 | Q9 | Total score (9%) |
| --- | --- | --- | --- | --- | --- | --- | --- | --- | --- | --- |
| JOSEPHINE et al, 2018 | Y | Y | NA | Y | Y | Y | Y | Y | NR | 7 |
| Bayard et al, 2008 | Y | Y | Y | Y | Y | Y | Y | Y | Y | 9 |
| Derebe et al, 2020 | Y | Y | Y | Y | Y | Y | Y | Y | Y | 9 |
| Achille, et al, 2020 | Y | Y | Y | Y | Y | Y | Y | Y | Y | 9 |
| Deborah et al, 2020 | Y | Y | Y | Y | Y | Y | Y | NR | Y | 8 |
| Mustafa et al, 2023 | Y | Y | Y | Y | Y | Y | Y | Y | Y | 9 |
| Belay et al, 2023 | Y | Y | Y | Y | Y | Y | Y | Y | Y | 9 |
| Teferi et al, 2022 | Y | Y | Y | Y | Y | Y | Y | NR | Y | 8 |
| Victor et al, 2021 | Y | Y | Y | Y | Y | Y | Y | NR | Y | 8 |
| Taiwo et al, 2014 | Y | Y | Y | Y | Y | Y | Y | NR | Y | 8 |

**Key:** **Y**= Yes; **NR**= Not reported, **NA**=Not appropriate

**Question codes:**

1. Was the sample frame appropriate to address the target population?

2. Were study participants sampled in an appropriate way?

3. Was the sample size adequate?

4. Were the study subjects and the setting described in detail?

5. Was the data analysis conducted with sufficient coverage of the identified sample?

6. Were valid methods used for the identification of the condition?

7. Was the condition measured in a standard, reliable way for all participants?

8. Was there appropriate statistical analysis?

9. was the response rate adequate, and if not, was the low response rate managed appropriately?
